# Hippocampal 4-Hz oscillations emerge during stationary running in a wheel and are resistant to medial septum inactivation

**DOI:** 10.1371/journal.pone.0284514

**Published:** 2023-04-19

**Authors:** Ivan Alisson Cavalcante Nunes de Lima, Hindiael Belchior

**Affiliations:** 1 Bioinformatics Multidisciplinary Environment (BioME), Federal University of Rio Grande do Norte, Natal, RN, Brazil; 2 Department of Physical Education, Federal University of Rio Grande do Norte, Natal, RN, Brazil; Universita degli Studi di Palermo, ITALY

## Abstract

Recent studies described 2–4 Hz oscillations in the hippocampus of rats performing stationary locomotion on treadmills and other apparatus. Since the 2–4 Hz rhythm shares common features with theta (5–12 Hz) oscillations—such as a positive amplitude-running speed relationship and modulation of spiking activity—many have questioned whether these rhythms are related or independently generated. Here, we analyzed local field potentials and spiking activity from the dorsal CA1 of rats executing a spatial alternation task and running for ~15 s in a wheel during the intertrial intervals both before and after muscimol injection into the medial septum. We observed remarkable 4-Hz oscillations during wheel runs, which presented amplitude positively correlated with running speed. Surprisingly, the amplitude of 4-Hz and theta oscillations were inversely related. Medial septum inactivation abolished hippocampal theta but preserved 4-Hz oscillations. It also affected the entrainment of pyramidal cells and interneurons by 4-Hz rhythmic activity. In all, these results dissociate the underlying mechanism of 4-Hz and theta oscillations in the rat hippocampus.

## Introduction

The rodent hippocampus expresses abundant rhythmic activity across distinct and sometimes overlapping frequency ranges [1]. Theta (5–12 Hz) oscillations are the most prominent rhythm in the rat hippocampus. It rises whenever rats engage in locomotor activity or in response to sensory stimulus [2–4]. Theta amplitude positively correlates with running speed in mazes, open fields, and other apparatus like stationary running on the treadmill and running wheel or passive ambulation [5–13]. Pharmacological experiments established at least two types of hippocampal theta oscillations: an atropine-resistant theta (6–12 Hz, type I) present during locomotor behaviors, and an atropine-sensitive theta (5–9 Hz, type II) expressed in response to sensory stimulation [14, 15]. In addition, lesion or inactivation of the medial septum-diagonal band of Broca abolishes hippocampal theta and impairs memory performance [16, 17].

In contrast, oscillations at lower frequencies, as in the delta (1–4 Hz) band emerge during quiet behaviors and slow-wave sleep episodes but are typically suppressed during locomotor activity [2, 18]. The difference between associated behaviors and brain states expressing delta and theta oscillations gave rise to the idea that these two rhythms are essentially orthogonal [19]. However, recent studies have shown that sustained oscillatory activity in the 2–4 Hz also emerges during locomotion in stationary conditions, like treadmills, head-fixed and virtual reality apparatus [10, 12, 13]. Hippocampal 2–4 Hz oscillations present similarities with the concomitant theta rhythm, such as a positive relationship between its instantaneous power and running speed, and its phase-modulation of spiking activity [12, 13]. Due to these resemblances, many researchers have raised concerns about the interdependence between the concurrent 2–4 Hz and theta oscillations and suggested that they may not be clearly dissociated in two genuinely independent rhythms. To sort apart 2–4 Hz oscillations from the classical theta activity and untangle their underlying mechanisms, we analyzed the effects of muscimol injection into the medial septum over hippocampal oscillations during maze and wheel runs.

## Results

### 4-Hz oscillations emerge in the rat hippocampus during wheel running

**Fig 1:**
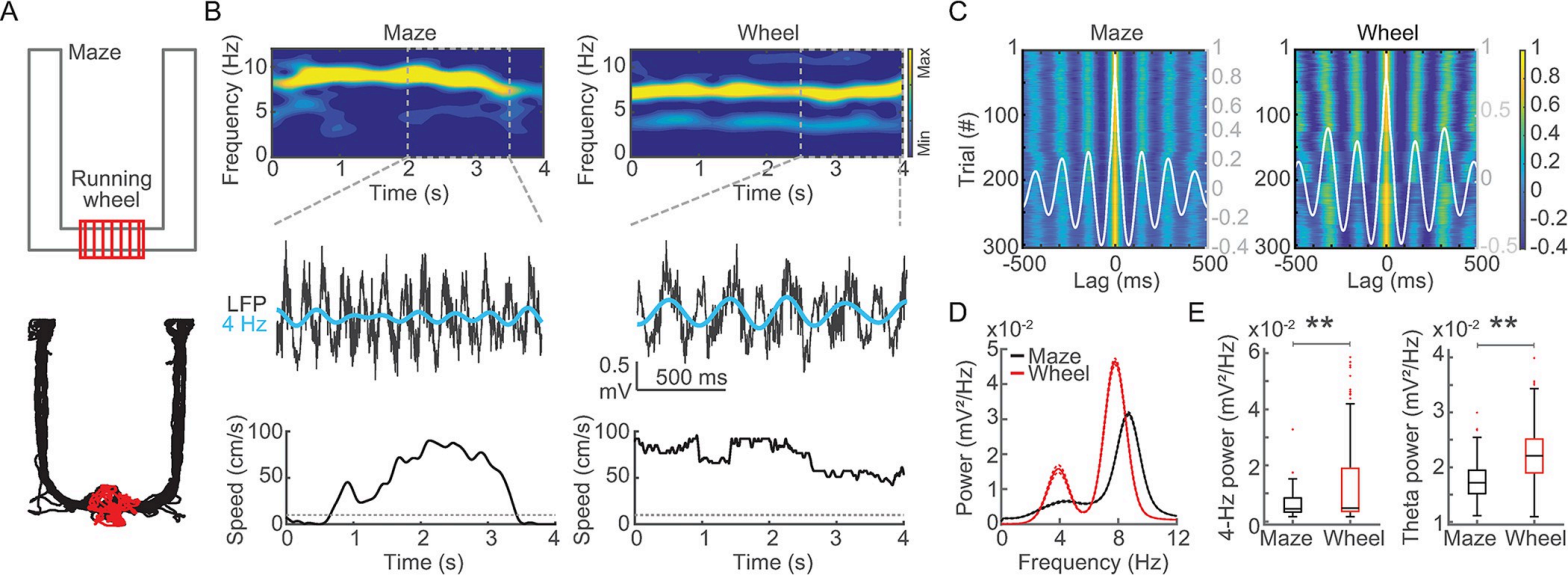
Hippocampal 4-Hz oscillations emerge during wheel but not maze running. (A) Schematic representation of the U-shaped maze (gray) coupled to a running wheel (red, upper). A typical example of the spatial trajectory of a rat on the maze (black) and in the wheel (red, lower). (B) Spectrograms showing energy at 0–12 Hz frequencies during representative maze and wheel runs (left and right, respectively, upper). Notice that only wheel running exhibits prominent energy at 4 Hz and 8 Hz frequencies. Raw LFP and 3–5 Hz band-filtered signals (gray and cyan, respectively, middle), and the instantaneous running speed at the same maze and wheel runs as above (lower). (C) Autocorrelograms of LFP signals across 304 runs at the maze and the wheel (left and right, respectively). Gray traces represent the average autocorrelograms across trials. Interpeak intervals were 145 ms (6.8 Hz) during maze runs and 320 ms (3.1 Hz) during wheel runs. (D) Average power spectra from LFP obtained during maze and wheel runs (black and red, respectively, n = 304 trials). Solid lines depict the mean and dashed lines depict ± SEM. (E) Boxplot showing the distribution of power in the 3–5 Hz band during maze and wheel runs (left, p < 0.01, WSR test). Distribution of power in the theta (6–10 Hz) band during maze and wheel runs (right, p < 0.01, WSR test). ** indicates p < 0.01 at the WSR test.

Rats performed a spatial alternation task on a U-shaped maze and ran for ~15 s on a wheel during the intertrial intervals (Fig 1A). Consistent with previous reports, the spectral decomposition of CA1 LFP showed prominent theta oscillations while rats ran on both maze and wheel (Fig 1B). Interestingly, however, only wheel runs further exhibited remarkable rhythmicity at 4-Hz. Hippocampal 4-Hz oscillations were noticeable at the raw LFP and spectrograms during wheel but maze runs, even in trials with similar running speeds (Fig 1B and S1A and S1B Fig). Autocorrelograms (ACG) revealed strong rhythmicity during maze and wheel runs with mean interpeak intervals of 145 ms (6.8 Hz) and 320 ms (3.1 Hz), respectively (Fig 1C). Average power spectra at the group level showed a single peak at 8.7 Hz during maze runs and two peaks at 7.8 Hz and at 4 Hz in the wheel (Fig 1D). The 4-Hz power was significantly higher during wheel than maze runs (Fig 1E, left), and its peak frequency was lower at the wheel (S2A Fig). The theta power was higher at the wheel (Fig 1E, right), while its peak frequency was slower at the wheel (S2B Fig).

### Hippocampal 4-Hz amplitude correlates with running speed in the wheel

**Fig 2:**
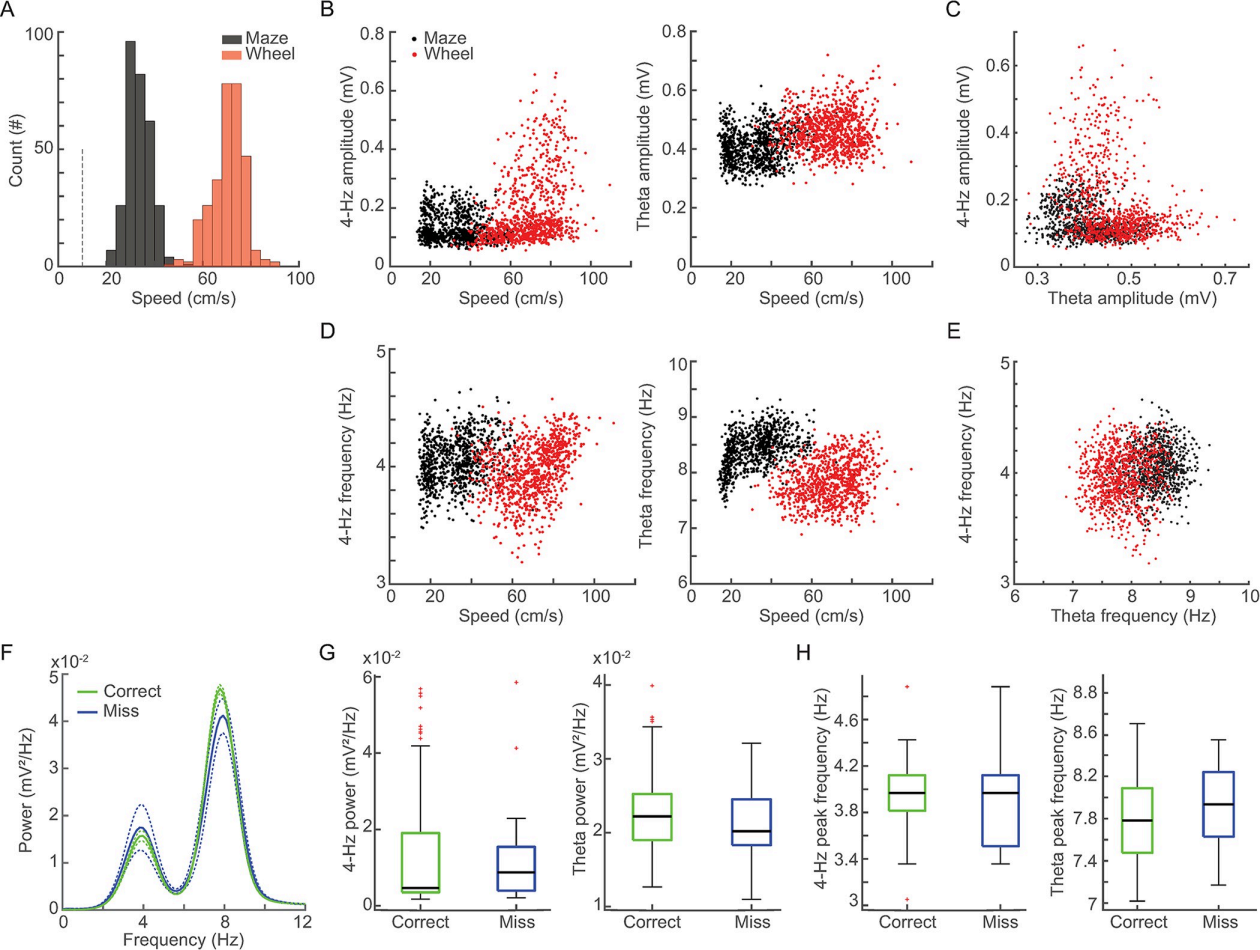
Hippocampal 4-Hz oscillations are modulated by running speed but are not affected by choice performance. (A) Histograms of running speeds at the maze (black) and at the wheel (red, n = 304 trials, p < 0.01, WRS test). (B) Scatter plots of running speed and the instantaneous 4-Hz amplitude (left, wheel: rho = 0.38, p < 0.01, maze: rho = -0.15, p < 0.01) and theta amplitude (right, wheel: rho = 0.15, p = 0.74, maze: rho = 0.01, p < 0.01). Notice the inverse relationship between 4-Hz amplitudes at maze and wheel conditions. (C) Relationship between 4-Hz and theta amplitude on the maze (black, rho = -0.31, p < 0.01) and in the wheel (red, rho = -0.28, p < 0.01). (D) Scatter plots show the relationship between running speed and the instantaneous 4-Hz frequency (left, 4-Hz at the wheel: rho = 0.38, p < 0.01; 4-Hz at the maze: rho = 0.27, p < 0.01) and theta frequency (right, theta at the maze: rho = 0.46, p < 0.01; theta at the wheel: rho = 0.23, p < 0.01). (E) Relationships between the 4-Hz and theta instantaneous peak frequency on the maze (black, rho = 0.11, p < 0.01) and in the wheel (red, rho = 0.10, p < 0.01). Similar results were obtained using 1 and 5 seconds windows. (F) Average power spectra during wheel run previous to correct (green, n = 286 trials) and incorrect (blue, n = 18 trials) choices at the spatial alternation memory task. Solid lines represent means and dashed lines represent ± SEM. (G) Boxplots of 4-Hz band power (left, p = 0.26, WRS test) and theta band power (right, p = 0.34, WRS test) during wheel runs previous to correct and incorrect choices. (H) Boxplots of 4-Hz peak frequency (left, p = 0.75, WRS test) and theta peak frequency (right, p = 0.36, WRS test) during wheel runs before correct and incorrect trials (see also histograms in S3 Fig).

Animals ran faster at the wheel (Fig 2A), which could suggest that 4-Hz oscillations are due to higher running speeds. In fact, the instantaneous 4-Hz amplitude and running speed were positively correlated at the wheel (Fig 2B, left), and negatively correlated at the maze. In turn, the theta amplitude was weakly correlated with maze speed but not with wheel speed (Fig 2B, right). The instantaneous amplitude of 4-Hz and theta oscillations were negatively correlated on both maze and wheel runs (Fig 2C). The peak frequency of 4-Hz and theta oscillations were positively correlated with running speed in both conditions (Fig 2D, left and right, respectively). The instantaneous peak frequency at 4-Hz and theta were weakly and positively correlated in both conditions (Fig 2E). Since 4-Hz oscillations were only observed during the intertrial intervals of the spatial alternation task, we next evaluated whether 4-Hz power and frequency in the wheel were associated with memory performance. The wheel runs previous to correct and incorrect choices exhibited similar power spectra (Fig 2F), in which neither 4-Hz nor theta band power nor peak frequency were statistically different previous to correct and incorrect choices (Fig 2G and 2H).

### Hippocampal 4-Hz oscillations are resistant to medial septum inactivation

**Fig 3:**
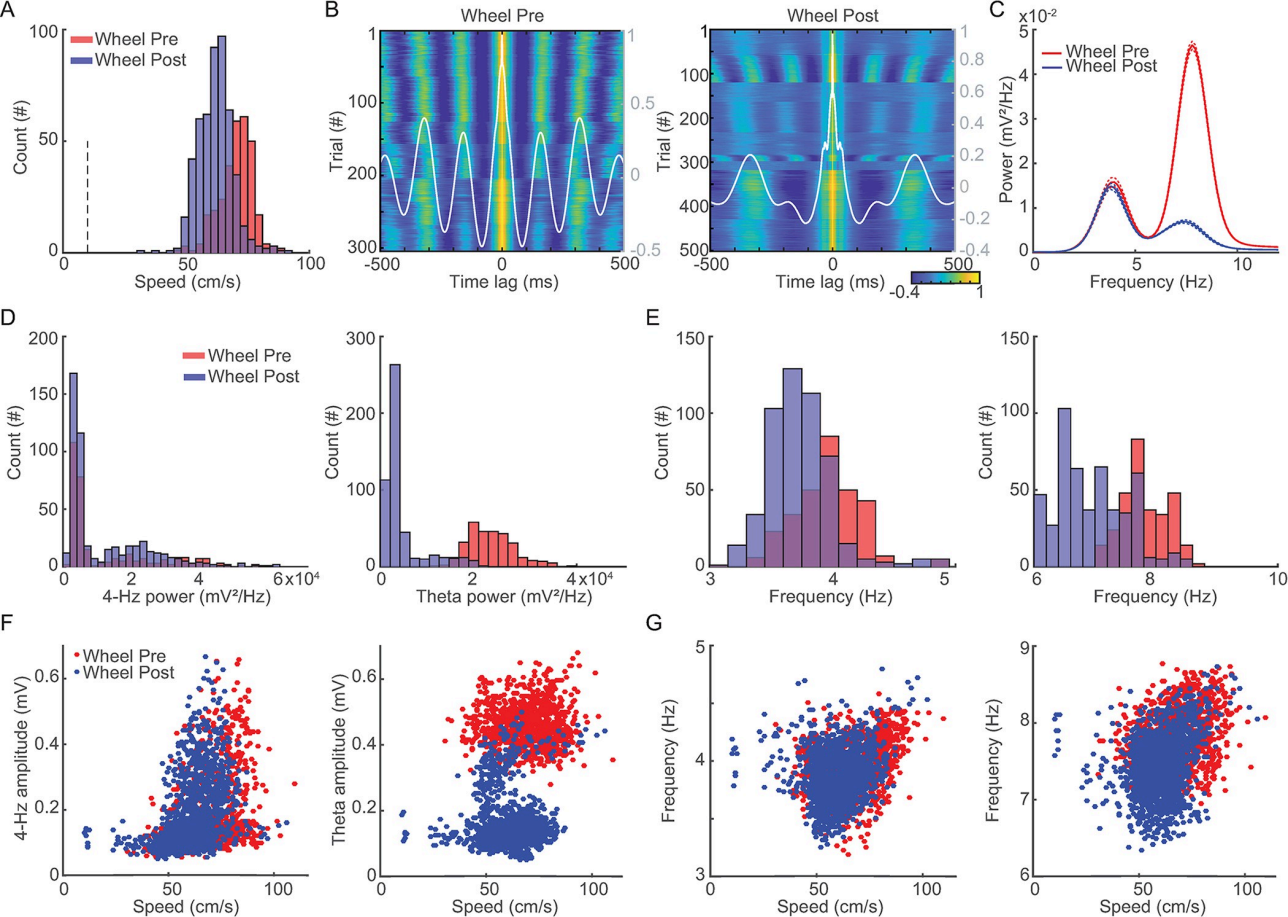
Hippocampal 4-Hz oscillations are resistant to pharmacological inactivation of the medial septum. (A) Histograms of running speed at the wheel before (red, n = 304 trials, 13.54 s) and after (blue, n = 501 trials, 13.43 s) muscimol inactivation of the medial septum (p < 0.01, WRS test). (B) Color-coded autocorrelograms of LFP signals across runs at the wheel before (left) and after (right) muscimol. Gray traces represent the average autocorrelograms. (C) Average power spectra at 0–12 Hz during wheel runs before (red) and after (blue) muscimol injection. Solid lines represent the mean and dashed lines represent ± SEM. (D) Histograms of 4-Hz band power (left, p = 0.68, WRS test) and theta band power (right, p < 0.01, WRS test) before and after muscimol. (E) Histograms of 4-Hz band peak frequency (left, p < 0.01, WRS test) and theta band peak frequency (right, p < 0.01, WRS test) before and after muscimol. (F) Scatter plots of running speed and the instantaneous amplitude of 4-Hz (left, Pre: rho = 0.38, p < 0.01; and Post: rho = 0.52, p < 0.01) and theta (right, Pre: rho = 0.01, p = 0.74; and Post: rho = 0.08, p < 0.01) oscillations before and after muscimol. (G) Scatter plots of running speed and the instantaneous frequency of 4-Hz (left, Pre: rho = 0.38, p < 0.01; and Post: rho = 0.19, p < 0.01) and theta (right, Pre: rho = 0.23, p < 0.01; and Post: rho = 0.29, p < 0.01) oscillations before and after muscimol.

Next, we evaluated how medial septum inactivation affects 4-Hz and theta oscillations in the wheel. Muscimol injection reduced the running speed on the wheel (Fig 3A), with no changes in the duration of wheel runs (Post: 13.43 s, p = 0.06, WRS test). It also impaired choice performance at the spatial alternation task (S3 Fig). Autocorrelograms and power spectral analyses show that muscimol injection abolished theta rhythmicity but surprisingly preserved 4-Hz oscillations (Fig 3B and 3C; S1C and S1D Fig). Muscimol injections did not change 4-Hz power and significantly decreased theta power (Fig 3D, left and right, respectively). In addition, muscimol injections reduced both 4-Hz and theta peak frequency (Fig 3E, left and right, respectively).

We then evaluated whether the effects of medial septum inactivation on 4-Hz and theta oscillations were dependent on running speed in the wheel. Muscimol injection did not affect the 4-Hz amplitude-running speed relationship, while reduced theta amplitude (Fig 3F, left and right, respectively; S4 Fig). Noteworthy, we also found that muscimol injection increased 4-Hz power and reduced theta power when analyzing subsets of trials matched for similar wheel speeds (S5 Fig). Moreover, muscimol injection did not affect peak frequency, neither at 4-Hz nor at theta oscillations (Fig 3G, left and right, respectively). Muscimol abolished the relationship between 4-Hz and theta instantaneous amplitudes, but not instantaneous peak frequencies, in the wheel (S6A and S6B Fig, respectively).

### 4-Hz oscillations modulate the spiking activity of interneurons and pyramidal cells

**Fig 4:**
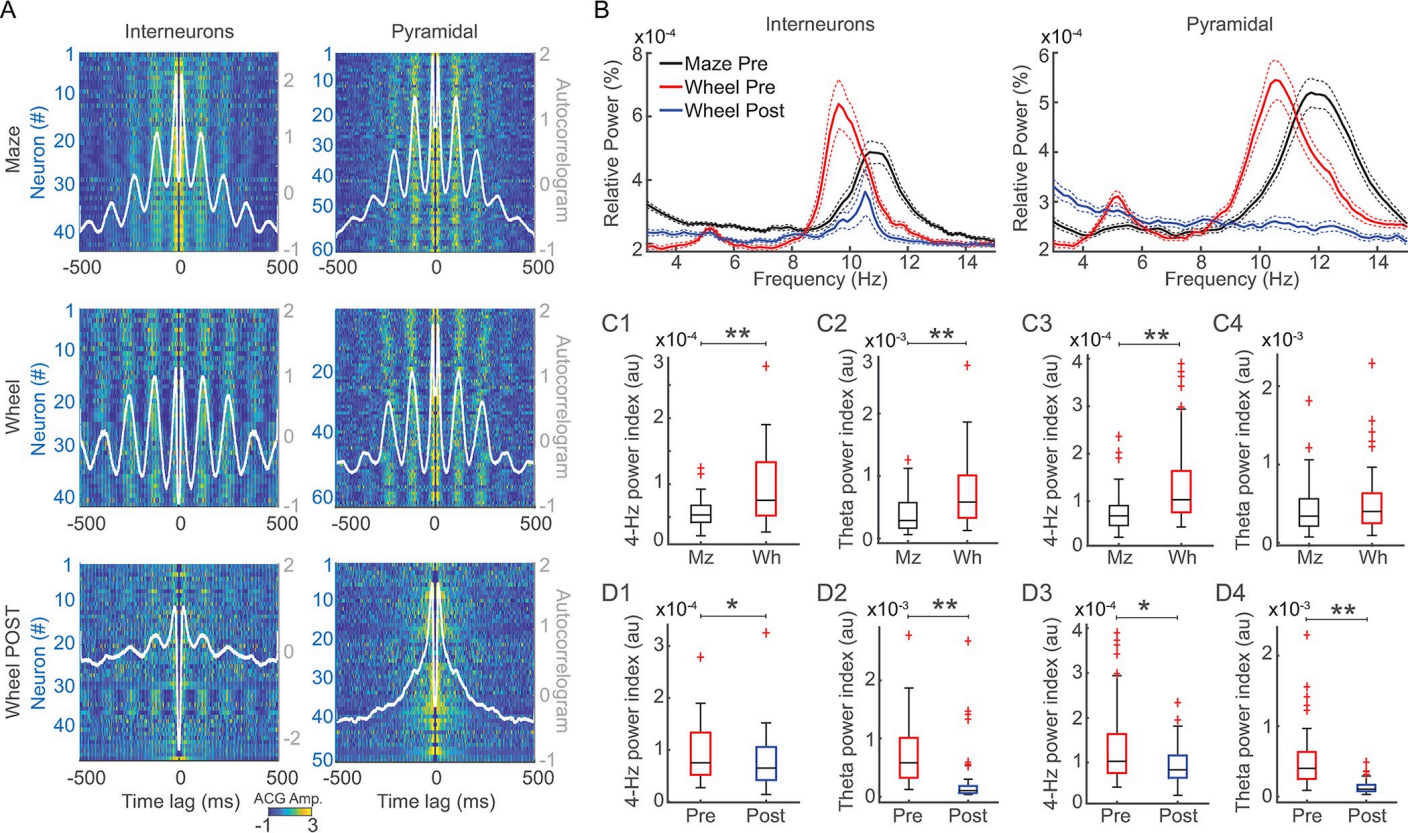
4-Hz rhythmicity of spiking activity decreases after medial septum inactivation. (A) Color-coded autocorrelograms of interneurons (left) and pyramidal cells (right) during maze runs (upper), and during wheel runs before (middle) and after muscimol injection (lower). White lines depict the average ACG for the neuronal population. Notice that neuronal rhythmicity of both cell types increased during wheel runs and strongly decreased after muscimol injection. Interneurons interpeak intervals at 4-Hz in the wheel: 246 ms; on the maze: 227 ms (p < 0.01, WRS test). Interneurons ACG amplitude at 4-Hz in the wheel: 0.94; on the maze: 0.73 (p < 0.01, WRS test). Pyramidal cells interpeak intervals at 4-Hz in the wheel: 232 ms; on the maze: 218 ms (p < 0.01, WRS test). Pyramidal cells ACG amplitudes in the wheel: 0.66; on the maze: 0.5 (p < 0.01, WRS test). Interneurons interpeak intervals at 4-Hz in the wheel before: 0.94; after muscimol: 0.9 (p = 0.42, WRS test). Interneurons ACG amplitudes at 4-Hz in the wheel before: 246 ms; after muscimol: 250 ms (p = 0.37, WRS test). Pyramidal cells interpeak intervals at 4-Hz in the wheel before: 232 ms; after muscimol: 232 ms (p = 0.94, WRS test). Pyramidal cells ACG amplitudes at 4-Hz in the wheel before: 0.66; after muscimol: 0.5 (p < 0.01, WRS test). (B) Relative (%) power spectral density of interneurons’ (left) and pyramidal cells‘ (right) spiking activity during maze runs (black), wheel runs before (red), and wheel runs after muscimol injection (blue). (C) Boxplots comparing spike PSDs of interneurons (left, C1-2) and pyramidal cells (right, C3-4) on 4-Hz and theta bands during maze and wheel runs. (D) Same as in panel C for wheel runs before (Pre) and after (Post) muscimol injection. * indicates p < 0.05, and ** indicate p < 0.01 at WRS or WSR tests, as described.

We evaluated autocorrelograms and power spectra of the spiking activity of putative interneurons and pyramidal cells during maze and wheel runs. Corroborating previous studies, autocorrelograms of spiking activity during maze runs presented strong rhythmicity in the theta band (Fig 4A, upper panel). During wheel runs, however, spike ACG exhibited remarkable rhythmicity in the 4-Hz band frequency (Fig 4A, middle panels). In this condition, interneurons presented longer interpeak intervals and higher amplitudes at 4-Hz than maze runs. Pyramidal cells also showed longer interpeak intervals and higher ACG amplitudes in the 4-Hz band frequency during wheel than maze runs. Muscimol injection significantly reduced firing rates of interneurons on both maze and wheel runs (p = 0.0004 and p = 0.0012, respectively, WSR test), but did not affect their ACG amplitude nor interpeak intervals at 4-Hz (Fig 4A, lower panels, S7A and S7B Fig, respectively). In turn, muscimol did not significantly change firing rates of pyramidal cells at the maze nor the wheel (p = 0.11 and p = 0.07, respectively, WSR test), while reduced the ACG amplitude but not the interpeak interval at 4-Hz of pyramidal cells (S7A and S7B Fig, respectively).

Power spectra of interneurons’ and pyramidal cells’ spikes during maze runs exhibited a single peak in the theta band frequency (Fig 4B, left and right, respectively). In contrast, wheel runs exhibited two peaks: one in the theta band and a second peak around 4-Hz. Interneurons and pyramidal cells showed a higher power index at 4-Hz during wheel than maze runs (Fig 4C1 and 4C3, respectively). In contrast, only interneurons, not pyramidal cells, presented a higher power index at theta during wheel than maze runs (Fig 4C2 and 4C4, respectively). Finally, muscimol injection significantly reduced interneurons’ and pyramidal cells’ power index in the 4-Hz (Fig 4D1 and 4D3) and in the theta bands (Fig 4D2 and 4D4).

## Discussion

We report the emergence of 4-Hz oscillations in the dorsal CA1 of rats engaged in stationary running in a wheel. Hippocampal 4-Hz oscillations directly correlate with wheel speed and entrain rhythmic spiking activity of pyramidal cells and interneurons. Unlike theta rhythms, 4-Hz oscillations are resistant to pharmacological inactivation of the medial septum. Our results disentangle the mechanisms of generation of 4-Hz and theta oscillations in the rat hippocampus.

Current evidence has shown that during stationary locomotion on treadmills, virtual reality, and head-fixed apparatus the rat hippocampus can simultaneously exhibit two steady rhythms within the 1–12 Hz frequency range: the classical 5–12 Hz theta and a new 2–5 Hz oscillation [10, 12, 13]. Our findings confirm the concurrency of hippocampal 4-Hz and theta oscillations during stationary runs, extending to the wheel apparatus.

Interestingly, only 4-Hz amplitude displayed a positive relationship with running speed in the wheel (rho = 0.31). We found that theta and 4-Hz instantaneous amplitudes were inversely related (rho = -0.28), which differed from previous reports using a virtual reality apparatus [13]. These results are at least partially consistent with Furtunato et al. (2020) that described an inverse relationship between theta and 2–4 Hz amplitudes, in which theta amplitudes decreased and 2–4 Hz amplitudes increased across consecutive runs at the same speed (30 cm/s) on a treadmill [12]. Despite the concomitant occurrence, the orthogonality between these rhythms may indicate a potential dissociation in the mechanism of generation of hippocampal theta and 4-Hz oscillations.

To evaluate this possibility, we tested whether pharmacological inactivation of the medial septum through intracerebral microinjections of muscimol affected hippocampal 4-Hz oscillations. As expected [20, 21], muscimol injections abolished hippocampal theta. However, hippocampal 4-Hz oscillations were resistant to medial septum inactivation and preserved a positive relationship with running speed in the wheel. These results provide the first evidence of different mechanisms of generation of 4-Hz and theta oscillations that co-occur in the rat hippocampus during stationary runs.

It is still unclear why 4-Hz oscillations are expressed in stationary locomotion but not usually observed during translational running conditions. Previous studies presented preliminary data on speed-modulated 4-Hz during wheel runs [5, 7]. However, none of them directly compared stationary versus translational locomotion. Here we observed in the same recordings that only wheel runs were accompanied by 4-Hz oscillations but not speed-matching maze runs. It confirms previous findings comparing runs in a linear track and in a virtual reality apparatus [13]. In addition, since our behavioral protocol tested wheel runs during the intertrial intervals of a spatial alternation task, we were able to evaluate if 4-Hz oscillations in the wheel were associated with choice performance. Neither the power nor the peak frequency at 4-Hz in the wheel differed before correct and incorrect choices, suggesting that 4-Hz oscillations were not associated with the cognitive demands of the spatial alternation task.

Theta oscillations dominate the hippocampus during translational locomotion, with amplitude and frequency positively associated with running speed and acceleration [22, 23]. When rats engage in stationary running, however, the absence of linear body movements could theoretically decouple vestibular and proprioceptive signals. Safaryan and Mehta (2021) proposed that it could in turn dissociate hippocampal theta in two components at 4 and 8 Hz [13]. Alternatively, others have shown that hippocampal oscillations may also synchronize with the respiratory rhythm [10, 24]. Chi et al. (2016) showed that mice running in head-fixed conditions exhibited long periods of steady respiration around 4-Hz that entrain brain oscillations at the same rhythm in the dentate gyrus [10]. The respiratory frequency is more stable during stationary than translational locomotion because of the reduction of bouts of sniffing, which could also explain why 4-Hz oscillations are not observed during maze runs. Future research that simultaneously monitors breathing and hippocampal rhythms in stationary and translational runs could investigate this hypothesis.

It is also unknown whether 4-Hz oscillations observed during stationary runs relate to the slower theta oscillations typically at frequencies around 5 Hz observed spontaneously or in response to stimuli in urethane-anesthetized rodents [15]. Current evidence however points in the contrary direction since atropine injections in the medial septum also abolish hippocampal theta oscillations in urethane-anesthetized rats [25]. In fact, Mofleh and Kocsis (2021) have found that theta-states of urethane-anesthetized rats exhibit two concurrent slow components: a slower 2-Hz oscillation that is coherent with respiratory activity, and a 5-Hz theta oscillation [26]. Despite the lower frequencies, we believe these results resemble our findings in awake rats running in the wheel. Additionally, alternative approaches that block or stimulate theta oscillations—such as electrical and optogenetic manipulations—could also be useful to further disentangle the mechanisms of 4-Hz and theta oscillations. In consonance with this, Bland et al. (2016) used electrical stimulation of the medial raphe nucleus to abolish type-II sensory processing-related theta oscillations in the hippocampus while leaving type-I movement-related theta unchanged in wheel-running rats [27]. These results suggest that type-I and type-II theta oscillations are at least partially generated by independent mechanisms.

Finally, the present work provides pharmacological and electrophysiological evidence that hippocampal 4-Hz and theta oscillations are independently generated during stationary locomotion.

## Materials and methods

The dataset used in this study was previously acquired at the Pastalkova Lab on the Janelia Research Campus [28] and made available at http://datadryad.org/ under a public domain dedication license.

### Behavioral and electrophysiological recordings

Two 64-channel linear silicon probes (Neuronexus or Janelia RC) were bilaterally implanted at the dorsal CA1 area (coordinates: -4.0 mm AP, ± 3 mm ML) of the rat hippocampus (n = 3 animals across ten sessions). Local field potentials (LFP), spiking activity, and digital video recordings were obtained during a delayed spatial alternation memory task in a U-shaped maze coupled to a running wheel in which animals ran (~15 s) during the intertrial intervals. Electrophysiological and behavioral recordings were obtained before (n = 304 trials) and after (n = 501 trials) muscimol microinjections into the medial septum. Detailed experimental procedures can be found in previous publications [21, 27].

### Data analysis

All data analyses were performed using custom-made and built-in routines in MATLAB (MathWorks, Natick, MA). First, LFP recordings were notch-filtered between 55 Hz and 65 Hz to remove 60 Hz electrical noise. LFP signals were visually inspected to detect time intervals presenting electrical or movement artifacts. Next, time intervals in which the amplitude of LFP signals was larger than two times the standard deviation were excluded from further analysis (only recordings from rat A943 presented artifacts). Epochs presenting locomotion speed on the maze and in the wheel higher than 10 cm/s were further analyzed.

We used the “eegfilt” function (EEGLAB Toolbox, [29]) to obtain 4-Hz (3–5 Hz) and theta (6–10 Hz) components of the LFP signals. We refer to 4-Hz oscillations, the spectral frequency between 3–5 Hz. The “pwelch” function from the Signal Processing Toolbox was used to obtain the power spectral density of LFP signals and spiking activity (1-s window length with 90% overlap, Fig 1D). The “hilbert” function from the Signal Processing Toolbox was used to obtain the instantaneous amplitude, phase, and frequency of 4-Hz and theta band components. The “xcorr” function (0.5-s window length, option type “coeff”, Figs 1C and 3B) from the Signal Processing Toolbox was used to obtain the autocorrelograms of LFP signals (Figs 1C and 3B). The “spectrogram” function (2-s window length with 90% overlap, Fig 1B) from the Signal Processing Toolbox to obtain the time-frequency decomposition.

The rhythmicity of interneurons’ and pyramidal cells’ spiking activity was estimated through autocorrelograms (ACG) and power spectral densities (PSD) of spike times. Only neurons with an average firing rate larger than 1 Hz during maze and wheel runs were further analyzed. We used the “xcorr” function (0.5-s window length, option type “coeff”, Fig 4A) to calculate the ACG of each neuron individually. The ACG of each neuron was normalized before obtaining the group result: NormACG = [ACG—min(ACG)] / [max(ACG)—min(ACG)], where max(ACG) and min(ACG) denote the maximum and minimum ACG values of each neuron, respectively. Only neurons with normalized ACG larger than a threshold value of 0.2 were further analyzed. To obtain the group result, the normalized ACGs were then averaged by cell type and condition (Fig 4A). To graphically display the ACG of each neuron (Fig 4A), we used a color-coded plot of the z-scored data smoothed by 100 points, in which warm colors mean higher ACG values for a given neuron at a given time lag. The values of ACG peak amplitudes and interpeak intervals of each neuron were obtained within specific time windows for 4-Hz (lags at 200–300 ms) and theta bands (lags at 100–200 ms).

Similarly, we used the “pwelch” function (5-s time window, and no overlap, Fig 4B) to estimate the PSD of each neuron individually. Only the neurons previously analyzed in the ACG were included in PSD analyses. The relative PSD of each neuron was obtained before computing the group result: relative PSD = [PSD / sum(PSD)], in which sum(PSD) denotes the sum of power across frequency values. The group result shown in Fig 4B was obtained by averaging the relative PSD by cell type and condition. Similarly to previous studies [21], the spike PSD peaks were at higher frequencies than previously observed in LFP PSD (Fig 1D). We thus adjusted the 4-Hz and theta frequency bands to fit the oscillatory spiking activity for 4–6 Hz and 8–15 Hz, respectively. Since different conditions exhibited different levels of baseline power, we calculated the power index to compare across conditions. For instance, the 4-Hz power index = max(PSD value at 4–6 Hz band)—PSD value at 6 Hz, in which max(PSD) denotes the maximum power value within the band of interest.

### Statistical analysis

All statistical analyses were performed in MATLAB. An alpha level of 0.05 was used to denote statistical significance. In the figures, one and two asterisks denote p < 0.05 and p < 0.01, respectively. Group data are expressed as mean ± standard error of the mean (SEM) or median and quartiles over trials, as indicated. We used the Shapiro-Wilk test to evaluate data normality. The Wilcoxon signed-rank (WSR) test or paired t-test was used to compare maze and wheel running conditions. The Wilcoxon rank-sum (WRS) test or Student’s t-test was used comparing wheel runs before and after muscimol injections, and between wheel runs previous to correct and incorrect choices. The “corr” function (option type “Spearman”) from the Statistics and Machine Learning Toolbox was used to obtain the Spearman’s rank-based correlation coefficients (rho) between speed-amplitude and speed-frequency at the 4-Hz and theta bands (5-s time window, Fig 2B and 2D, for instance), and also between the instantaneous amplitude of coexisting 4-Hz and theta oscillations (5-s time window, Fig 2C and 2E, for instance).

## Supporting information

S1 Fig Raw LFP signals and 4-Hz oscillations during maze and wheel runs before and after muscimol injection. Representative examples of raw LFP (black) and 4-Hz-filtered signals (cyan) recorded during maze and wheel runs before (A and B, respectively) and during maze and wheel runs after (C and D, respectively) muscimol administration. The upper, middle, and lower panels depict LFP signals recorded from three different rats (rat A498, rat A543, and rat A943, respectively). Only epochs of running speed larger than 10 cm/s are shown.
(TIF)

S2 Fig Peak frequency within 4-Hz and theta bands during maze and wheel runs. (A) Peak frequency within the 4-Hz band during maze and wheel runs (left, p < 0.01, WSR test, n = 304 trials). (B) Peak frequency within the theta band during maze and wheel runs (right, p < 0.01, WSR test, n = 304 trials). ** indicate p < 0.01 at the WSR test.
(TIF)

S3 Fig Power and peak frequency in 4-Hz and theta bands during wheel runs according to choice performance on the task before and after muscimol injection in the medial septum. (A) Percentage of correct choices before (Pre, correct 94.07%, green, from 304 trials) and after (Post, correct 60.27%, green, from 501 trials) muscimol administration into the medial septum (across 10 sessions from 3 animals, p < 0.01, WRS test). (B) The absolute number of correct (green) and incorrect (blue) choices before (Pre, correct 286 trials) and after muscimol injection (Post, correct 302 trials). (C) Histograms of 4-Hz band power (left) and theta band power (right) during wheel runs previous to correct (green) and incorrect (blue) choices. (D) Distribution of 4-Hz peak frequency (left) and theta peak frequency (right) during wheel runs before correct (green) and incorrect (blue) trials.
(TIF)

S4 Fig Relationship between running speed and the instantaneous amplitude of 4-Hz and theta oscillations before and after muscimol injections. (A) Scatter plots of running speed and the instantaneous amplitude of 4-Hz oscillations before and after muscimol from individual rats. The upper, middle, and lower panels show data from rats A498, A543, and A943, respectively. The individual rho- and p-values are A498: Pre, rho = 0.39, p < 0.01; Post, rho = 0.41, p < 0.01. A543: Pre, rho = 0.25, p < 0.05; Post, rho = 0.57, p < 0.01. A943: Pre, rho = 0.36, p < 0.01; Post, rho = 0.08, p = 0.062. (B) Scatter plots of running speed and the instantaneous amplitude of theta oscillations before and after muscimol. A498: Pre, rho = 0.38, p < 0.01; Post, rho = 0.17, p < 0.01. A543: Pre, rho = 0.53, p < 0.01; Post, rho = 0.53, p < 0.01. A943 Pre, rho = 0.03, p = 0.057; Post, rho = 0.12, p < 0.01.
(TIF)

S5 Fig Effects of muscimol injections on 4-Hz and theta oscillations during wheel runs when controlling for variations in running speed. (A) Speed distributions in subsets of wheel runs ranging from 55 cm/s to 63 cm/s before (red) and after (blue) muscimol injection (p = 0.12, WRS test; Pre: n = 39 trials, and Post: n = 196 trials). (B) Average power spectra at 0–12 Hz during wheel runs before (red) and after (blue) muscimol injection in subsets of trials matched for running speed. Solid lines represent the mean and dashed lines represent ± SEM. (C) Boxplots showing the distribution of power in the 3–5 Hz band during wheel runs before and after muscimol injections in the same subsets of trials as before (p < 0.01, WRS test). (D) Distribution of power in the theta (6–10 Hz) band during wheel runs before and after muscimol injections in the same subsets of trials as before (p < 0.01, WRS test). ** indicate p < 0.01 at the WRS test.
(TIF)

S6 Fig Relationship between 4-Hz and theta instantaneous amplitude and frequency before and after muscimol injection. (A) Relationship between 4-Hz and theta amplitude in the wheel before (Pre, red, rho = -0.28, p < 0.01, n = 908, 5-s bins) and after (Post, blue, rho = -0.04, p = 0.08, n = 1540, 5-s bins) muscimol injection. (B) Relationship between 4-Hz and theta peak frequency in the wheel before (Pre, red, rho = 0.10, p < 0.01, n = 908, 5-s bins) and after muscimol injection (Post, blue, rho = 0.19, p < 0.01, n = 1540, 5-s bins).
(TIF)

S7 Fig Amplitude and interpeak intervals of spike ACGs at 4-Hz in the wheel before and after muscimol injection. (A) ACG amplitudes of interneurons’ (left, p < 0.05, WRS test) and pyramidal cells’ (right, p < 0.01, WRS test) spikes at the 4-Hz frequency range. (B) ACG interpeak intervals of interneurons’ (left, p = 0.82, WRS test) and pyramidal cells’ (right, p = 0.53, WRS test) spikes at the 4-Hz frequency range. Red and blue bars depict wheel runs before and after muscimol injections, respectively.
(TIF)
