# Supplementary figures and images for: Hippocampal 4-Hz oscillations emerge during stationary running in a wheel and are resistant to medial septum inactivation
Source: PLoS One. 2023 Apr 19;18(4):e0284514. doi: 10.1371/journal.pone.0284514 (PMC10115258; doi:10.1371/journal.pone.0284514)

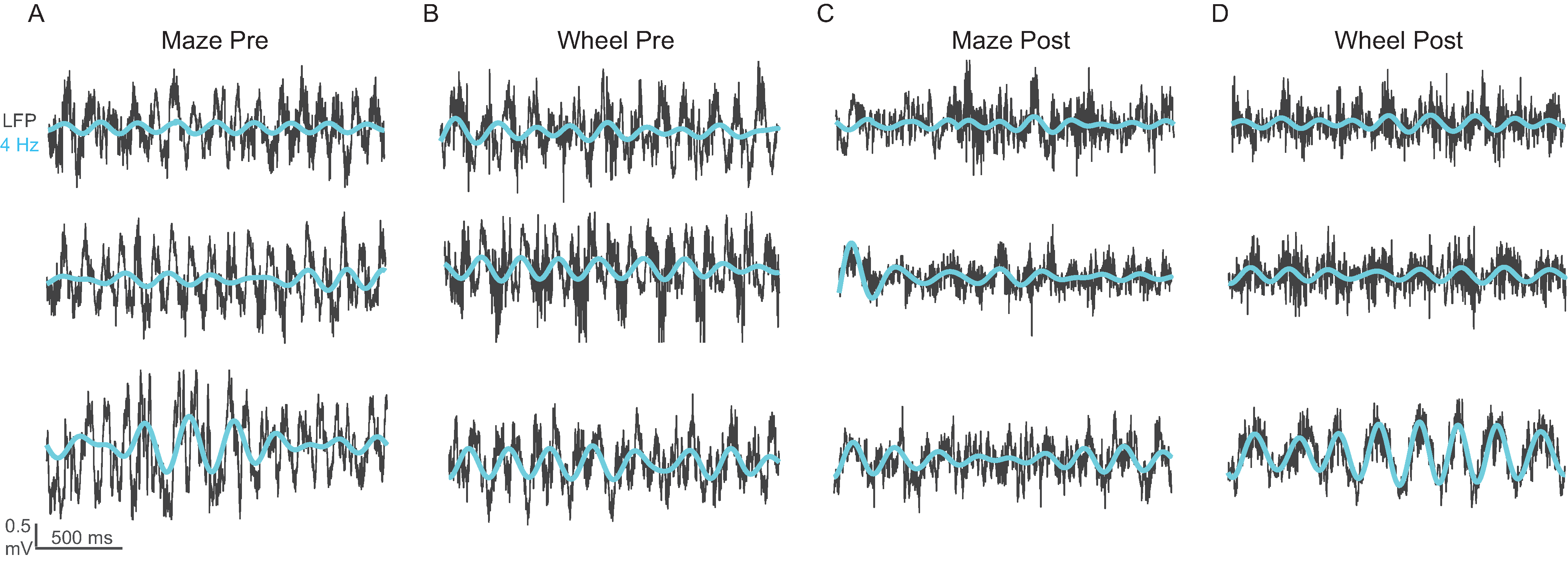

Supplement: S1 Fig — Representative examples of raw LFP (black) and 4-Hz-filtered signals (cyan) recorded during maze and wheel runs before (A and B, respectively) and during maze and wheel runs after (C and D, respectively) muscimol administration. The upper, middle, and lower panels depict LFP signals recorded from three different rats (rat A498, rat A543, and rat A943, respectively). Only epochs of running speed larger than 10 cm/s are shown. (TIF) [file pone.0284514.s001.tif]

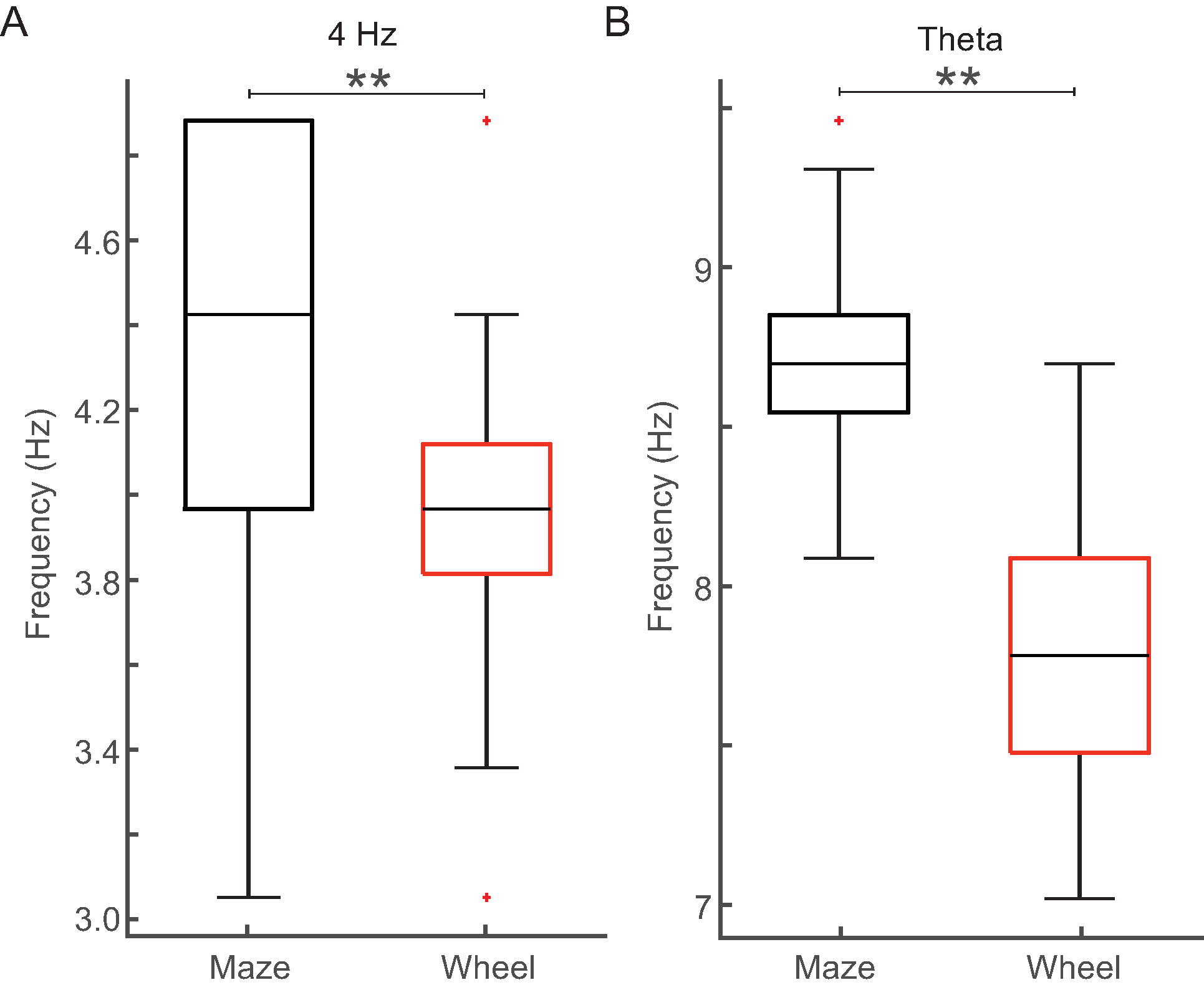

Supplement: S2 Fig — (A) Peak frequency within the 4-Hz band during maze and wheel runs (left, p < 0.01, WSR test, n = 304 trials). (B) Peak frequency within the theta band during maze and wheel runs (right, p < 0.01, WSR test, n = 304 trials). ** indicate p < 0.01 at the WSR test. (TIF) [file pone.0284514.s002.tif]

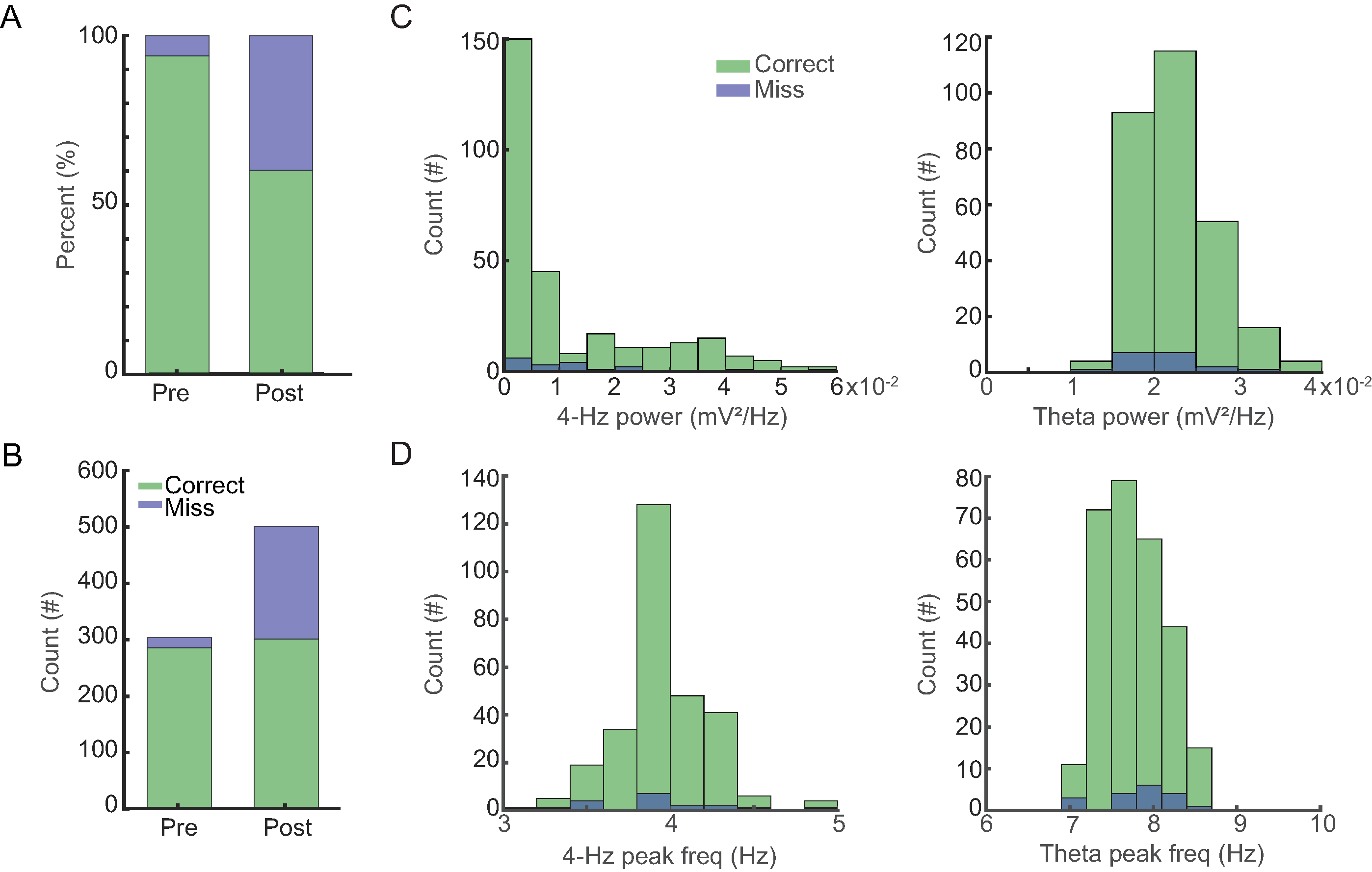

Supplement: S3 Fig — (A) Percentage of correct choices before (Pre, correct 94.07%, green, from 304 trials) and after (Post, correct 60.27%, green, from 501 trials) muscimol administration into the medial septum (across 10 sessions from 3 animals, p < 0.01, WRS test). (B) The absolute number of correct (green) and incorrect (blue) choices before (Pre, correct 286 trials) and after muscimol injection (Post, correct 302 trials). (C) Histograms of 4-Hz band power (left) and theta band power (right) during wheel runs previous to correct (green) and incorrect (blue) choices. (D) Distribution of 4-Hz peak frequency (left) and theta peak frequency (right) during wheel runs before correct (green) and incorrect (blue) trials. (TIF) [file pone.0284514.s003.tif]

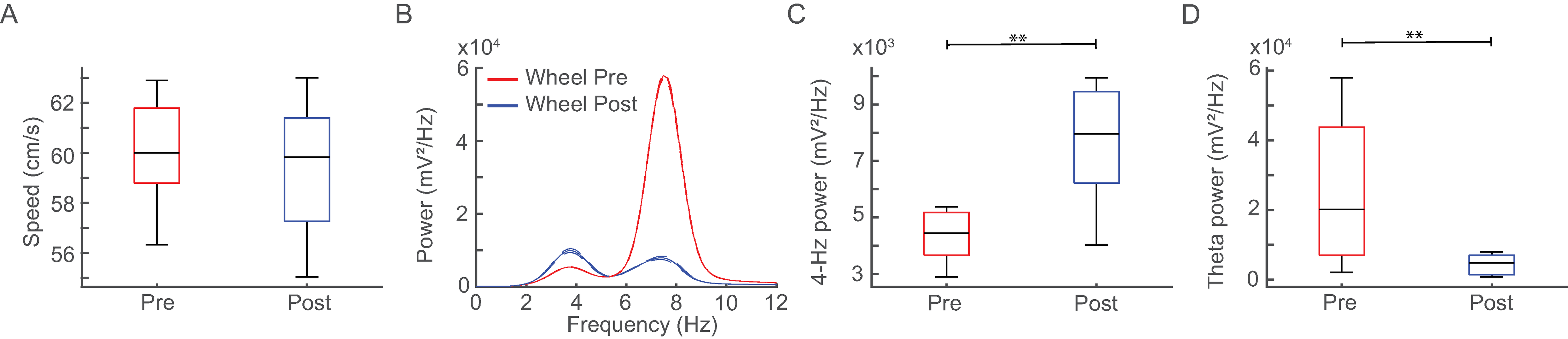

Supplement: S4 Fig — (A) Scatter plots of running speed and the instantaneous amplitude of 4-Hz oscillations before and after muscimol from individual rats. The upper, middle, and lower panels show data from rats A498, A543, and A943, respectively. The individual rho- and p-values are A498: Pre, rho = 0.39, p < 0.01; Post, rho = 0.41, p < 0.01. A543: Pre, rho = 0.25, p < 0.05; Post, rho = 0.57, p < 0.01. A943: Pre, rho = 0.36, p < 0.01; Post, rho = 0.08, p = 0.062. (B) Scatter plots of running speed and the instantaneous amplitude of theta oscillations before and after muscimol. A498: Pre, rho = 0.38, p < 0.01; Post, rho = 0.17, p < 0.01. A543: Pre, rho = 0.53, p < 0.01; Post, rho = 0.53, p < 0.01. A943 Pre, rho = 0.03, p = 0.057; Post, rho = 0.12, p < 0.01. (TIF) [file pone.0284514.s004.tif]

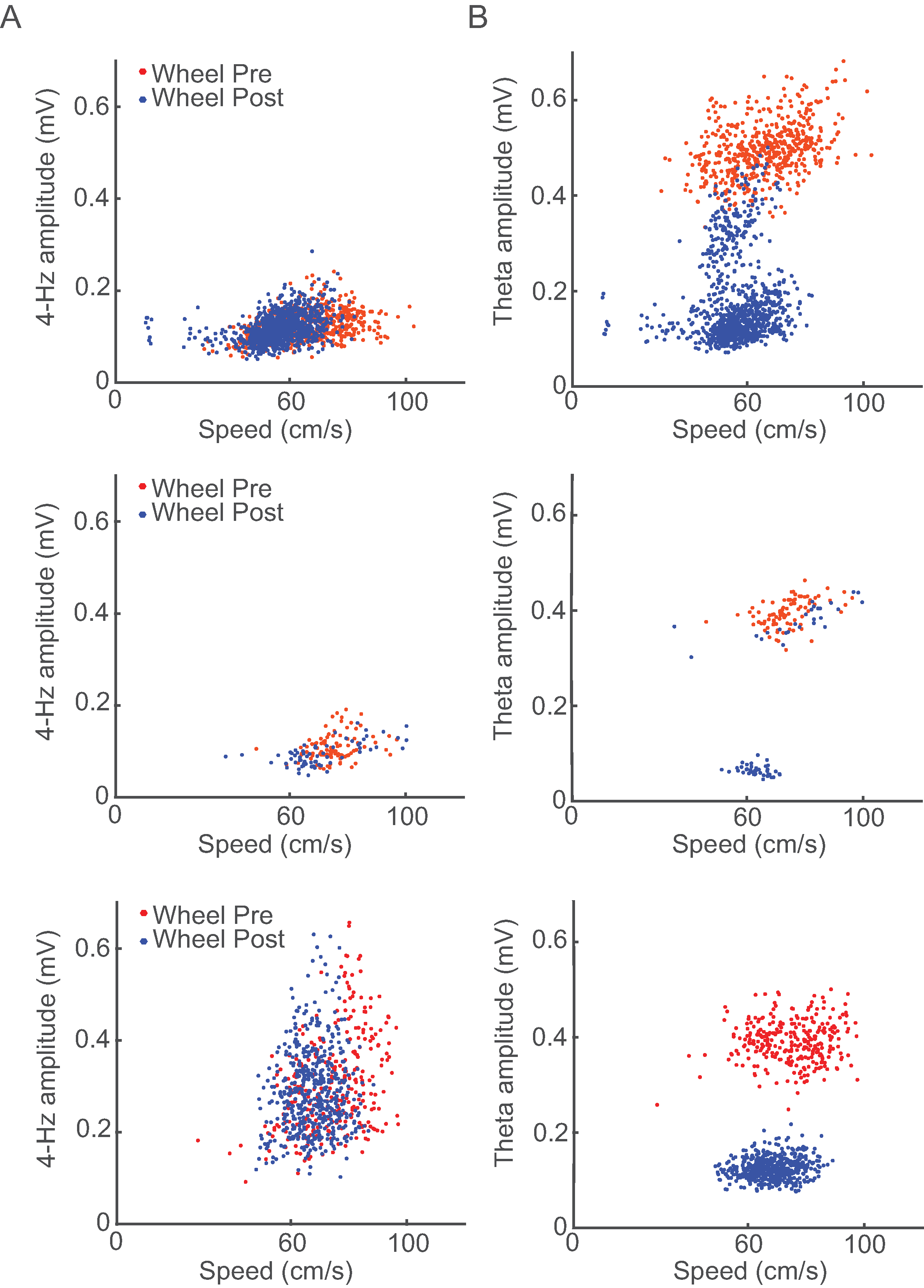

Supplement: S5 Fig — (A) Speed distributions in subsets of wheel runs ranging from 55 cm/s to 63 cm/s before (red) and after (blue) muscimol injection (p = 0.12, WRS test; Pre: n = 39 trials, and Post: n = 196 trials). (B) Average power spectra at 0–12 Hz during wheel runs before (red) and after (blue) muscimol injection in subsets of trials matched for running speed. Solid lines represent the mean and dashed lines represent ± SEM. (C) Boxplots showing the distribution of power in the 3–5 Hz band during wheel runs before and after muscimol injections in the same subsets of trials as before (p < 0.01, WRS test). (D) Distribution of power in the theta (6–10 Hz) band during wheel runs before and after muscimol injections in the same subsets of trials as before (p < 0.01, WRS test). ** indicate p < 0.01 at the WRS test. (TIF) [file pone.0284514.s005.tif]

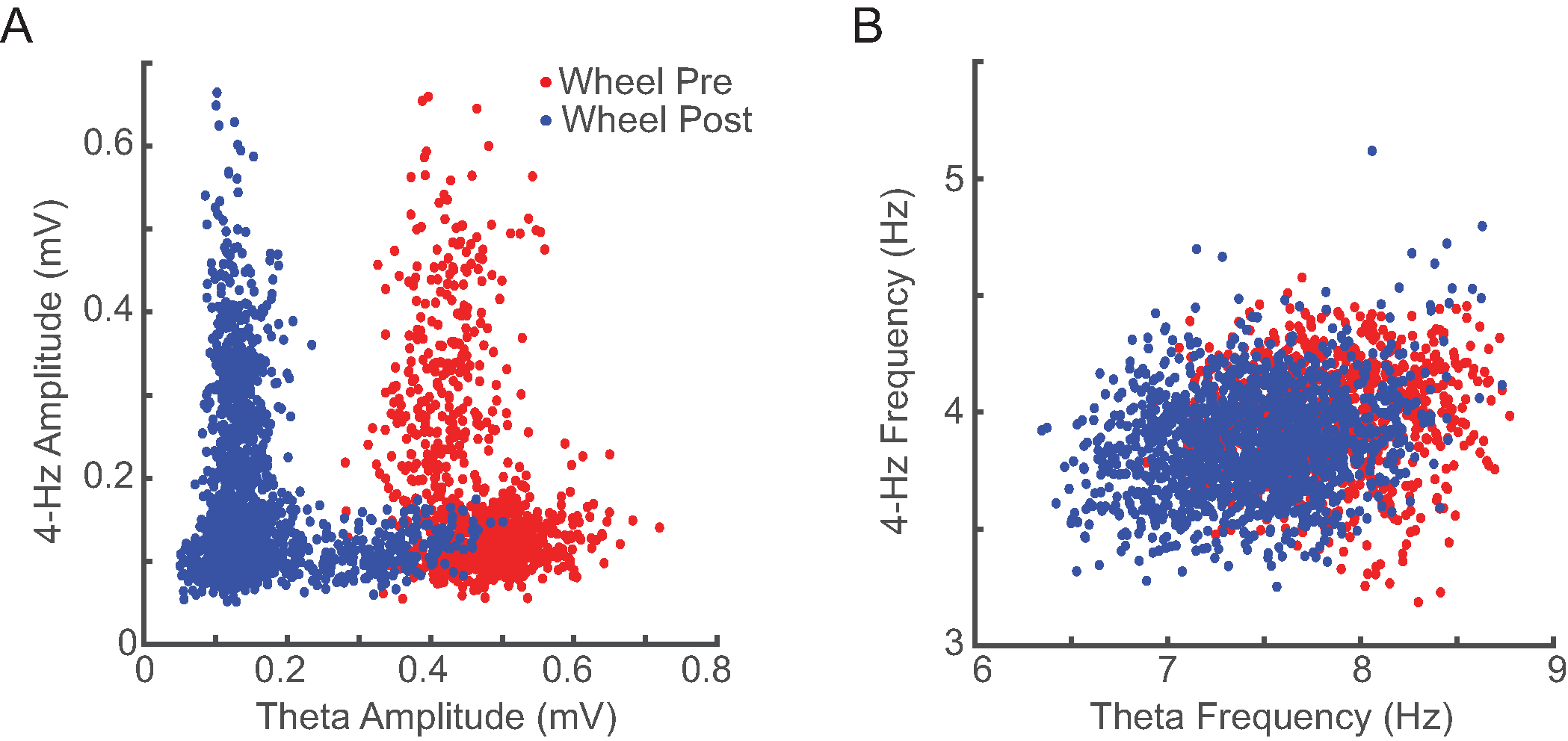

Supplement: S6 Fig — (A) Relationship between 4-Hz and theta amplitude in the wheel before (Pre, red, rho = -0.28, p < 0.01, n = 908, 5-s bins) and after (Post, blue, rho = -0.04, p = 0.08, n = 1540, 5-s bins) muscimol injection. (B) Relationship between 4-Hz and theta peak frequency in the wheel before (Pre, red, rho = 0.10, p < 0.01, n = 908, 5-s bins) and after muscimol injection (Post, blue, rho = 0.19, p < 0.01, n = 1540, 5-s bins). (TIF) [file pone.0284514.s006.tif]

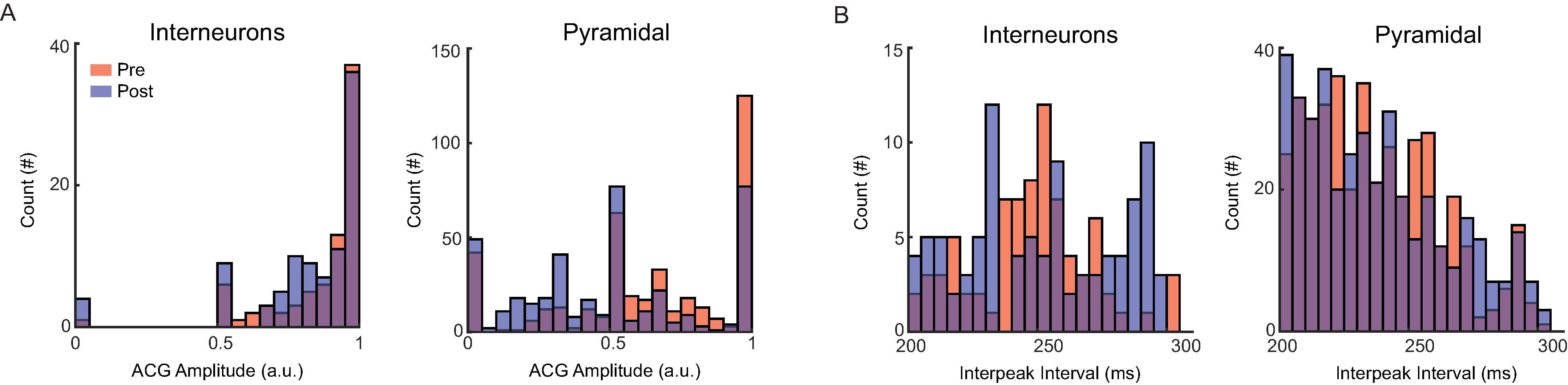

Supplement: S7 Fig — (A) ACG amplitudes of interneurons’ (left, p < 0.05, WRS test) and pyramidal cells’ (right, p < 0.01, WRS test) spikes at the 4-Hz frequency range. (B) ACG interpeak intervals of interneurons’ (left, p = 0.82, WRS test) and pyramidal cells’ (right, p = 0.53, WRS test) spikes at the 4-Hz frequency range. Red and blue bars depict wheel runs before and after muscimol injections, respectively. (TIF) [file pone.0284514.s007.tif]
